# Hematological Parameters, and Hematopoietic Growth Factors: Epo and IL-3 in Response to Whole-Body Cryostimulation (WBC) in Military Academy Students

**DOI:** 10.1371/journal.pone.0093096

**Published:** 2014-04-02

**Authors:** Zbigniew Szygula, Anna Lubkowska, Czesław Giemza, Anna Skrzek, Iwona Bryczkowska, Barbara Dołęgowska

**Affiliations:** 1 Institute of Human Physiology, University School of Physical Education, Krakow, Poland; 2 Department of Functional Diagnostics and Physical Medicine, Faculty of Health Sciences, Pomeranian Medical University in Szczecin, Szczecin, Poland; 3 Department of Physiology, Faculty of Natural Sciences, Szczecin University, Szczecin, Poland; 4 Faculty of Physiotherapy, University School of Physical Education in Wroclaw, Poland; 5 Department of Laboratory Diagnostics and Molecular Medicine, Pomeranian Medical University, Szczecin, Poland; University of Wuerzburg, Germany

## Abstract

The effects of extreme cold on the human body are not fully understood, there are also no reports on the effect of cryogenic temperatures on the levels of erythropoietin (EPO) and interleukin 3 (IL-3), two important factors that regulate hematopoiesis. ***Aim***: determination of changes in peripheral blood cell counts and EPO and IL-3 levels induced by a series of 10, 20 and 30 standard whole-body cryostimulation (WBC) treatments. The study involved 45 men, experimental group (EXP, n = 30) subjected to 30 WBC treatments (−130°C, treatment duration: 3 minutes) and a control group (CON, n = 15). Blood samples were collected before the series of treatments and after 10, 20 and 30 treatments. After 10 and 20 treatments we observed lower red blood cell counts and hematocrit and hemoglobin levels compared to baseline (p<0.05) and the control group (p<0.05). Additionally we observed an increase in hemoglobin concentration in plasma (p<0.05), and bilirubin after 10 and 20 treatments, and a decrease in plasma concentration of haptoglobin after 10, 20 and 30 treatments (p<0.05). The number of leukocytes was higher after 10 and 20 WBC treatments compared to baseline and the CON group. EPO concentration in plasma was elevated and the concentration of IL-3 was lower after 10, 20 and 30 WBC treatments. The decrease in indices of the erythrocytic system, plasma hemoglobin and bilirubin, with a simultaneous decrease in haptoglobin concentrations after 10 and 20 WBC treatments, may be due to increased intravascular hemolysis. At the same time there was a small, but statistically significant increase in the concentration of EPO stimulated erythropoiesis which could facilitate a return of erythrocytic system indices to initial levels after 30 WBC treatments. Changes in the white blood cell system showed transient mobilization of this system under the influence of WBC.

## Introduction

Recently, cryotherapy and cryostimulation have gained in popularity in the prevention and treatment of musculoskeletal overload in competitive sports and sports medicine, and also the rejuvenation of healthy individuals to accelerate recovery and increase resistance. The initial use of this type of physical therapy in the treatment of acute and chronic soft tissue trauma, pain relief and reduction of inflammatory process activity, expanded the use of cryogenic temperatures for post-operative care after orthopedic surgery, in rheumatoid and nervous system diseases (e.g. spastic paresis after stroke, multiple sclerosis, depressive syndromes, vegetative neurosis), or as an analgesic and relaxing agent for patients in the terminal stages of cancer [1]. Therefore, there are a lot of papers and articles reporting the effect of cryogenic temperatures on changes in the bodies of individuals with various diseases [2], [3], [4], [5], [6].

Unfortunately, the mechanism and the therapeutic effect of cryogenic temperatures on the human body is still not fully understood. Research is often conducted on small groups, with significant differences in procedures in which cryostimulation (or cryotherapy) is often used in combination with other forms of treatment or as part of physical training. This does not allow unambiguous interpretation of results to assess the impact of cryogenic temperatures on the human body. Therefore literature reports are often contradictory and inadequate. Moreover, very often changes observed after WBC are interpreted with reference to the results obtained after the application of temperatures close to zero, such as immersion or swimming in cold water [7], [8], [9].

Whole body cryostimulation (WBC) is an application of extremely low temperatures (from −100°C to −140°C) on the human body. The term “cryostimulation” is increasingly often used to highlight the stimulatory effect of cryogenic temperature, in contrast to cryotherapy where a therapeutic aim is emphasized.

It is believed that whole body cryostimulation in healthy individuals is a safe procedure and does not cause adverse changes in the body [10], and potential complications related with the treatment are very rare if the contraindications to the treatment are taken into account [1]. Furthermore, there are some reports on the favorable physiological phenomena resulting from exposure of the human body to cryogenic temperatures, such as overall improvement in well-being (relaxation, physical relaxation), analgesic effect, increase in muscle strength, profuse flow of blood in peripheral tissue, mobilization of the immunological system, increase in norepinephrine, testosterone (especially in men) and improvement in antioxidant capacity [1], [10], [11].

One very interesting issue is the effect of WBC on peripheral blood and hematological indicators. Changes in peripheral blood indices could influence exercise capacity and general resistance to infectious agents. However, reports on this subject are scarce and sometimes contradictory. There are several papers evaluating the impact of cryogenic temperature on peripheral blood counts in healthy people. Some reports show a decrease in erythrocytic indices [12], [13], [14], [15], [16] and an increase in the number of leukocytes [16], [17] while others describe a lack of changes in erythrocyte and/or white blood cell systems [13], [14], [15], [18], [19] and in the number of platelets [14], [16] after WBC.

Because cytokines are a large group of molecules regulating hematopoiesis and various processes such as cell proliferation and differentiation, inflammatory and immune responses and others, a number of studies have been published over the past years on the effects of WBC on these molecules. The most commonly cytokines determined in these studies include IL-1α, IL-1β, IL-2, IL-6, IL-8, IL-10, IL-12 and TNF [8], [17], [19], [20], [21], [22]. However, none of the works to date have studied systemic cryotherapy on changes in the level of erythropoietin (EPO) and IL-3, important factors regulating hematopoiesis. As the effects of EPO an increase of the red blood cells mass, hematocrit and hemoglobin in the peripheral blood were observed, and consequently the transfer of oxygen to the peripheral tissues would be enhanced. Due to mentioned effects erythropoietin became a doping agent used by athletes particularly in endurance sports [23].

Interleukin-3 is a multipotent hematopoietic growth factor, stimulating hematopoiesis by the induction of proliferation and differentiation of early pluripotent stem cells and progenitor myeloid stem cells – myeloid, erythroid and megakaryocytic lineages. An administration of IL-3 to patients increased the number of leukocytes (primarily neutrophils, lymphocytes and eosinophils), as well as reticulocytes and platelets [24], [25].

As shown in previous studies, the effect of cryotherapy may depend on the number of treatments applied [17], [21], [26], [27], so the aim of this study was to investigate the effect of repeated exposure to the cryogenic temperatures used in a series of standard 10, 20 and 30 WBC treatments (−130°C, 3 min) on changes in key peripheral blood cell counts and levels of erythropoietin (EPO) and interleukin 3 (IL-3), i.e cytokines having a significant effect on hematopoiesis.

## Material and Methods

### Subjects

The research involved forty-five healthy male volunteers who had never been subjected to any form of cryotherapy.

The subjects were students of the General Tadeusz Kosciuszko Military Academy of Land Forces. Each participant gave a written assent before participating in the study, according to the Declaration of Helsinki. The study was approved by the local Ethics Committee (Ethics Committee of the Pomeranian Medical University; Ref.KB-0012/54/10). In addition, the superiors of the respondents agreed to include the WBC cryotherapy series into the daily schedules of the participants.

All subjects took part in initial medical qualification in order to eliminate those with potential contraindications to WBC. All the subjects were healthy and normotensive, with a body mass index (BMI) of between 21.12 and 26.3.

The men were randomly divided into two groups: an experimental group of 30 (EXP) and a control group of 15 (CON). The groups were homogeneous in terms of age (students of the same year, aged about 23 years), diet and daily physical activity, due to the scheduled joint activities, accommodation and food provided by the Military Academy. None of them participated in any kind of sports training. The characteristics of the study group are shown in Table 1. Participants of the experimental group (EXP) were exposed to a thirty treatment sessions of whole body cryostimulation (once a day) at an extremely low temperature in a cryogenic chamber (Creator CR 2002, Poland) at the premises of the University School of Physical Education in Wrocław, Poland. During the experiment, participants were required to not change the duration or intensity of their daily training and diet.

**Table 1:** Anthropometrical characteristics of the subjects (EXP – group of participants subjected to WBC, CON –control group).

	T_0_		T_3_	
	Before WBC		After 30th session	
	mean±SD		mean±SD	
Group	EXP	CON	EXP	CON
Age	23.5±0.85	23.6±0.9	23.5±0.85	23.6±0.9
Height [cm]	179.7±3.89	177.1±6.19	179.7±3.89	177.1±6.19
Body weight [kg]	79.1±8.04	78.8±5.3	78.1±9.0	78.1±7.3
BMI [kg/m^2^]	25±2.13	24.7±2.35	25±2.0	24.8±3.25

BMI – body mass index.

### Cryostimulation procedure

The treatment sessions were performed every day at the same morning hours, five days a week, from Monday to Friday. The subjects entered the chamber in groups of five persons. Each cryostimulation session lasted 3 minutes (−130°C). Entry to the cryo-chamber was preceded by a 30-second adaptation period in the vestibule at a temperature of −60°C, from which the subjects went further to the proper chamber, where they moved slowly in a circle, one after the other, without mutual contact, no additional movement or talking. After a minute, a change in the direction of motion was recommended. Contact with the participants was maintained via a camera in the room and voice contact. Before each treatment systolic and diastolic blood pressures were measured to monitor one of the contraindications to cryostimulation, i.e. high blood pressure.

Glasses, contact lenses and all jewelry were removed before entry to the chamber, as well as a thorough drying of the body to eliminate the sensation of cold, noses and mouths were secured with a surgical mask. During the cryostimulation procedure, the subjects were dressed only in shorts, socks, wooden clogs, gloves and a hat covering the auricles against frostbite.

### Blood sampling and analysis

During the experimental period, venous blood samples were collected four times to perform hematological and biochemical analyses. Blood was taken in the morning before the first treatment session (T0), in the morning of the day after the 10th treatment session (T1), and after the 20th (T2) and 30th treatment session (T3). To avoid changes in plasma volume and to ensure the proper hydration of the organism, our subject drank 15 ml of water per kg of body mass between 6 p.m. and 10 p.m. a day before blood collection. Blood samples were always obtained after overnight fasting, between 6.00 and 7.30 a.m., after 10 minutes rest in a sitting position, from an antecubital forearm vein using vacutainer tubes with an appropriate K2 EDTA anticoagulant (Sarstedt, Germany), separately into two tubes; one to determine blood counts (1.2 ml anticoagulated with 1 g/L EDTA) and the other for biochemical analysis of plasma (5 ml EDTA).

Directly after blood sampling, the following indicators were determined: the number of erythrocytes (RBC), hemoglobin concentration (Hb), hematocrit value (Ht), red blood cell indices: MCV - mean corpuscular volume, MCH - mean corpuscular hemoglobin, MCHC - mean corpuscular hemoglobin concentration, RDW - red cell distribution width, number of leukocytes, including the share of lymphocytes (LYM), granulocytes (GRA) and monocytes (MON), and the number of thrombocytes (PLT). Hematological parameters were measured by a hematology analyzer (HORIBA ABX Micros 60). Intra-assay precision and accuracy for all hematologic parameters were less than 1.5% and inter-assay precision and accuracy for all hematologic parameters were less than 2.0%. In plasma, the following indicators were measured: the concentration of extra cellular hemoglobin [g/dL] determined spectrophotometrically using Drabkin's reagent; haptoglobin (HAP) concentration [g/L] (CORMAY haptoglobin); bilirubin [mg/dL] (BIOLABO); and levels of IL-3, and erythropoietin (R&D Systems Quantikine ELISA kits). The coefficients of variation of both the intra-assay and inter-assay were less than 10%. The minimum detectable concentration for IL3 was 7.4 pg/mL and less than 0.6 mU/mL for Quantikine IVD ELISA Human Epo.

### Statistical analysis

The normality of distribution of dependent variables was tested with the Shapiro-Wilk test. Since in some cases the data distribution was not normal, nonparametric statistical analyses were conducted. Comparison between before and after cryostimulation measurements were done with repeated-measures nonparametric Friedman's ANOVA and Wilcoxon rank sign tests. For comparison between two independent means, the Mann-Whitney U test was used. Correlations between observed variables were calculated by Spearman's coefficient of rank correlation. A p<0.05 was considered statistically significant for all tests. Each studied parameter was characterized by: sample size, arithmetic mean/median, standard deviation. All statistical calculations were conducted using the Statistica 6.0 package.

## Results

Anthropometrical characteristics of the participants are presented in Table 1. There were no differences in anthropometrical values between the two groups of subjects during the whole experiment.

The hematological profiles of the participants of both groups before (T_0_) and after the series of sessions of whole-body cryostimulation treatment (T_1_–T_3_) are presented in Table 2.

**Table 2:** Hematological profile of the participants (EXP  = E – group of participants subjected to WBC, CON –control group) after the series of whole-body cryostimulation treatment.

Parameters	T_0_ - Before WBC		T_1_ - After 10 session		T_2_ - After 20 session		T_3_ - After 30 session	
Group	EXP	CON	EXP	CON	EXP	CON	EXP	CON
	T_0E_	T_0C_	T_1E_	T_1C_	T_2E_	T_2C_	T_3E_	T_3C_
RBC	5.1±0.32	5.1±0.36	4.8±0.33 * ^T^_0E_	5.1±0.3* ^T^_1E_	4.89±0.27* ^T^_0E_	5.1±0.27* ^T^_2E_	5±0.34	5.1±0.28
[10∧12/]	(4.2–5.6)	(4.5–5.7)	(4.0–5.2)	(4.4–5.6)	(4.1–5.3)	(4.5–5.5)	(4.2–5.6)	(4.6–5.5)
Hb	15.1±0.74	15.2±0.46	14.4±0.94* ^T^_0E_	15.2±0.42* ^T^_E_	14.5±0.71* ^T^_0E_	15±0.36* ^T^_2E_	15.1±1.1	15.1±0.42
[g/dL]	(13.3–17.1)	(14.7–16.1)	(12.9–17.2)	(14.7–15.9)	(13.4–16.8)	(14.5–15.8)	(12.1–17.4)	(14.7–16)
Ht	46±2.2	46±2.6	42±2.7* ^T^_0E_	45±2.5* ^T^_1E_	42±3.1* ^T^_0E_	45±2.2* ^T^_2E_	45±3.0	45±2.8
[L/L]	(41–52)	(42–50)	(37–49)	(41–48)	(36–46)	(40–48)	(38–49)	(42–50)
MCV	89.7±2.4	90.1±2.6	88.8±2.0	89.4±2.6	89.6±2.0	89.6±2.3	90.4±2.4	91.4±2.4
[μm^3^]	(85.0–94.0)	(85.0–94.0)	(85.0–93.0)	(85.0–94.0)	(86.0–94.0)	(86.0–94.0)	(86.0–95.0)	(88.0–95.0)
MCH	29.2±1.0	28.8±1.2	29.6±0.9	29.4±1.0	29.7±0.9	29.6±0.9	29.7±1.2	29.7±1.1
[fmoL]	(27.5–31.3)	(26.6–30.5)	(27.9–31.6)	(27.9–31.1)	(28.1–31.7)	(28.6–31.1)	(28.2–33.9)	(27.9–31.8)
MCHC	34.9±0.7	32.1±0.53	33.3±0.5	32.9±0.4	33.2±0.4	33.1±0.3	32.8±0.9	32.4±1.1
[mmol/L]	(32.1–34.2)	(31.2–33.3)	(31.3–34.2)	(32.2–33.4)	(31.6–37)	(32.7–33.7)	(31.6–35)	(31.2–35)
RDW	13.4±0.4	13.3±0.6	13.7±0.4	13.3±0.6	14.2±0.5* ^T^_0E_	13.6±0.6* ^T^_2E_	13.5±0.52	13.3±0.5
[%]	(12.7–14.3)	(12.6–15.0)	(12.9–14.8)	(12.6–15.1)	(13.3–15.5)	(13.0–14.3)	(12.7–15.1)	(12.4–15)
Leucocytes	6.1±1.23	6.0±1.2	6.7±0.99* ^T^_0E_	6.1±1.5 * ^T^_1E_	6.8±0.86* ^T^_0E_	5.9±1.2* ^T^_2E_	6.5±0.91	6.0±2.0
[10∧^9^/L]	(4.2–8.3)	(4.5–8.0)	(4.3–9.4)	(4.4–8.9)	(4.1–7.4)	(3.9–8.7)	(4.4–7.8)	(3.7–9.9)
LYM	36.8±7.1	38.4±7.1	39.6±7.6	38.1±9.5	40.7±5.6* ^T^_0E_	38.3±7.0* ^T^_2E_	37.38±7.9	36.5±7.3
[%]	(24.8–42.2)	(23.0–44.7)	(29.9–45.9)	(18.7–47.3)	(36.5–45.4)	(25.8–41.4)	(23.3–42.9)	(26.2–48.2)
MON	4.9±1.0	5.0±1.0	5.4±1.0* ^T^_0E_	4.9±0.6	5.6±0.78* ^T^_0E_	4.9±0.7* ^T^_2E_	5.6±1.02* ^T^_0_	4.8±0.9** ^T^_3E_
[%]	(3.1–8.2)	(3.5–8.0)	(3.9–6.9)	(3.6–6.9)	(3.8–7.1)	(3.8–6.8)	(3.8–7.9)	(3.5–6.5)
GRA	55.6±7.1	56.4±7.4	58.4±8.1* ^T^_0E_	57.1±9.8	59.6±7.8* ^T^_0E_	53.7±7.2	56.8±8.2	58.2±9.3
[%]	(42.9–70.6)	(44.3–72)	(43.0–74.3)	(44.4–71.3)	(42.7–63.3)	(44.3–69.4)	(44.4–72)	(41.5–77.0)
PLT	236.4±44.2	228.9±42.7	252.7±36.3	250.3±60.1	232±38.4	229.5±38.1	224±49.3	221.3±32.1
[10∧^9^/L]	(167–314)	(167–314)	(157–326)	(159–297)	(156–310)	(170–307)	(152–326)	(165–287)

*statistically significant difference at p≤0.05;

**statistically significant difference at p≤0.01; RBC – red blood cells; HB – hemoglobin; HCT- hematocrit; MCV - mean corpuscular volume; MCH - mean corpuscular hemoglobin; MCHC - mean corpuscular hemoglobin concentration; RDW - red blood cell distribution width; WBC – white blood cells; LYM - lymphocytes; MON – monocytes; GRA – granulocytes; PLT – thrombocytes.

Red blood cells count, hemoglobin concentration and hematocrit dropped significantly after 10 sessions of WBC and remained decreased after 20 sessions in the experimental group (EXP) compared to the resting values (T_0E_), as well as to the values observed in the control group (CON). There were no changes in mean corpuscular volume (MCV), mean corpuscular hemoglobin (MCH) and mean corpuscular hemoglobin concentration (MCHC) in either group during the whole period. However, there was a rise in red cell distribution (RDW) in the EXP group after 20 sessions of WBC indicating an increased variation in the size of individual erythrocytes (anisocitosis).

A significant increase in leucocyte counts was observed after 10 and 20 WBC treatments compared to the initial values and values noted in control group. The percentage of lymphocytes increased after 20 sessions of WBC (p<0.05), the percentage of monocytes was significantly elevated after 10, 20 and 30 WBC treatments, while the percentage of granulocytes was higher after 10 and 20 WBC sessions.

An intensified hemolysis, as confirmed by significantly elevated levels of extraerythrocyte hemoglobin (plasma hemoglobin) and bilirubin as well as by decreased levels of haptoglobin (HAP), was observed after 10 and 20 WBC sessions compared to initial values and values observed in the control group (Table 3). Haptoglobin concentration was still depressed after 30 WBC treatments but extracellular hemoglobin was normal and bilirubin lowered after 30 WBC sessions (p<0.05).

**Table 3:** Biochemical parameters of blood plasma of participants (EXP  = E – group of participants subjected to WBC, CON –control group) during after series of whole-body cryostimulation treatment.

Parameters	T_0_		T_1_		T_2_		T_3_	
Group	Before WBC		After 10 session		After 20 session		After 30 session	
	EXP	CON	EXP	CON	EXP	CON	EXP	CON
	T_0E_	T_0C_	T_1E_	T_1C_	T_2E_	T_2C_	T_3E_	T_3C_
HB extracellular	26.30±7.2	25.67±15.73	31.47±6.1	26.0±6.5	29.86±9.2	28.4±0.21	30.73±5.7	30.2±12.2
[g/dL]			***T_0E_	*** T_1E_	***T_0E_			
HAP	0.34±0.03	0.35±0.06	0.23±0.01	0.34±0.07	0.22±0.03	0.33±0.23	0.32±0.06	0.36±0.05
[g/L]			***T_0E_	** T_1E_	***T_0_	*T_2E_	***T_0E_	*T_3E_
Bilirubin	1.24±0.04	1.23±0.51	1.34±0.07	1.23±0.56	1.41±0.07	1.28±0.9	1.19±0.09	1.13±0.82
[mg/dL]			*T_0E_	*T_1E_	*T_0E_	**T_2E_	*T_0E_	
EPO	299.4±31.33	304.05±34.26	312.9±39.31	309.50±37.49	331.65±28.12	316.00±28.05	329.60±45.64	305.75±31.16
[ng/ml]			**T_0E_	*T_1E_	***T_0E_	**T_2E_	**T_0E_	**T_3E_
IL-3	26.68±4.32	28.02±5.98	20.13±9.16	27.96±4.12	22.13±8.92	26.69±4.16	18.26±5.99	27.79±5.72
[pg/mL]				**T_1E_		**T_2E_	*T_0E_	**T_3E_

*statistically significant difference at p≤0.05;

**statistically significant difference at p≤0.01;

***statistically significant difference at p≤0.001; HB extracellular  =  plasma hemoglobin; HAP –haptoglobin.

Erythropoietin concentration was significantly elevated after 10, 20 and 30 WBC sessions when compared to the initial value and levels observed in the control group (Table 3). The most pronounced elevation of EPO was observed after 20 and 30 sessions. However, there have not been found any correlations between EPO and hematological parameters of blood in both groups.

Interleukin 3 (IL-3) decreased after 30 WBC sessions when compared to the initial level, however, concentration of this cytokine in plasma obtained from participants subjected to 10, 20 and 30 WBC was significantly lower compared to concentrations observed in the control group (Table 3).

## Discussion

The mechanisms of the effects of cryogenic temperatures on healthy people are not yet fully understood, although in the case of athletes, changes in peripheral blood may have a huge impact on the immune system (changes in the white blood cell system) or on exercise capacity (changes in the erythrocytes). In recent years there have been only a few reports providing data on the influence of extreme cold on healthy human hematological parameters and these reports are often contradictory and incomplete. Accordingly, we embarked on an investigation of the effects of different numbers of whole-body cryostimulation (WBC) treatments on peripheral blood cell counts and the levels of two cytokines (EPO and IL-3) which have a significant effect on hematopoiesis. To our knowledge this is the first report on the subject.

Erythropoiesis stimulating methods, such as high altitude training and hypoxic chambers, are commonly used in sport. Introduction of cryostimulation in sports medicine to enhance post-exercise recovery [10], [28] raises questions on the effects to the red blood cell system, and thus the oxygen capacity of blood and oxygen transported to tissues and organs. Can cryostimulation be used for the stimulation of erythropoiesis and if so - how much? Can the severity/magnitude of changes in peripheral blood be influenced by the number of whole-body cryostimulation treatments?

### Red blood cell system

After 10 WBC treatments involving active healthy young men, we found a significant decrease in hematological parameters such as the number of red blood cells, hemoglobin and hematocrit compared to baseline and to the values found in the control group. It was observed that these parameters were also similarly reduced after 20 WBC treatments, and after 30 treatments returned to baseline values and values similar to those of the control group. These results are consistent with most previous studies that have observed a significant decrease in the number of erythrocytes, hemoglobin and hematocrit after a series of five to 18 sessions of WBC [14], [15], [17], [18], [29]. Were the observed changes the result of increased intravascular hemolysis, prompted by the increased extracellular hemoglobin (plasma hemoglobin) and serum bilirubin and decreased plasma concentrations of haptoglobin found in this study in men undergoing ten and twenty WBC treatments? Plasma hemoglobin levels returned to normal only after 30 WBC treatments, and bilirubin and haptoglobin were reduced. There were no observed changes in these parameters in the men in the control group.

The increased hemolysis of erythrocytes under the influence of cryostimulation is also suggested by high values of plasma hemoglobin. Increases in plasma hemoglobin were also observed in another work, in blood taken 30 minutes after a single WBC treatment, with a concentration being almost four times higher than before treatment, and still higher the day after [30]. Vasoconstriction and muscle contraction (shivering) could be an answer. Loss of the older erythrocytes could be beneficial to stimulate RBC pool rejuvenation.

Banfi et al. argue, however, that WBC reduces hemolysis of erythrocytes in athletes [12], [13]. The basis for such a claim is, according to those authors, the lack of change in the number of erythrocytes, hematocrit and the share of reticulocyte, as well as increased levels of haptoglobin, mean sphered cell volume (MSCV), a decrease in mean reticulocyte volume (MRV) and immature reticulocyte fraction (IRF) [12], [13]. However, the result of that research seems to be insufficient to draw such definite conclusions since the authors did not measure intravascular hemolysis indexes such as plasma hemoglobin and bilirubin concentration. Moreover, the changes observed in that research cannot be compared with our results for at least three reasons.

Firstly, the study by Banfi et al. [12], [13] involved professional rugby players from the Italian National Rugby Team. Athletes have a larger pool of young red blood cells [31] which are more resistant to oxidative stress occurring during WBC, as demonstrated in other studies [32]. Secondly, when the rugby players were subjected to cryostimulation treatments they did not change their training program, exercising three hours a day, while the participants in our experiment, both in the experimental group and the control did not participate in any sports training. Thirdly, the rugby players were subjected to only five WBC sessions, while in this study, as well as by other authors who observed a marked reduction in the value of the aforementioned indicators, the number of WBC procedures ranged from 10 to 30 [14], [15], [16], [18]. As in other cases [12], [21], [26], [27] also here the depth and direction of the observed changes may result from the greater number of WBC treatments.

A similar relationship was observed by Lombardi et al. [15] among professional rugby players from the Italian National Rugby Team who were subjected to a greater number of treatments. After 14 sessions - two daily WBC for 7 consecutive days, they observed a decrease in RBC, hemoglobin, hematocrit, MCH and MCHC, which may indicate an increased hemolysis of erythrocytes, even in trained athletes, because plasma volume did not change significantly after a series of WBC treatments. In addition, Lombardi et al. [15] demonstrated an increased mean corpuscular volume (MCV) and red cell distribution width (RDW), which may indicate the occurrence of younger and a more diverse red blood cells in peripheral blood by stimulating erythropoiesis, although not too severely, as the percentage of RET remained unchanged. Similarly, a significant increase in RDW after 20 WBC treatments was observed in our study.

The erythropoiesis-stimulating effect of WBC treatment may also be indicated by changes in the hematological indices observed by Straburzyńska-Lupa et al. [18] in players of the Polish National Field Hockey team, who underwent 18 WBC treatments applied two times a day. The authors observed a decrease in RBC, hemoglobin and hematocrit, and a simultaneous increase in MCV, MCH and MCHC after a series of cryostimulation treatments. However, erythrocyte count and hematocrit returned to baseline values, and hemoglobin concentration exceeded the initial values, while the increase in MCV, MCH and MCHC was maintained for a week after the last treatment.

### White blood cell system

Information on the effects of cryogenic temperatures used during cryostimulation or whole-body cryotherapy on the white blood system in healthy people, are also very sparse, ambiguous and still do not sufficiently fully explain the observed changes.

On the basis of this study we can see stimulation of the white blood cells system in young, healthy people, as shown by a statistically significant increase in the number of white blood cells after 10 and 20 WBC treatments compared to baseline and the control group. An increase was also observed in the percentage of neutrophils and lymphocytes, being especially pronounced after 20 WBC treatments. The percentage of monocytes was elevated after 10, 20 and 30 treatments. Our previous reports also confirm that the series of cryostimulations resulted in a statistically significant increase in the total number of leukocytes after 10 [17] and 15 daily WBC treatments [16], although not exceeding clinical and laboratory norms. Additionally, the increase concerned also the number of lymphocytes, neutrophils and monocytes, and to a lesser extent eosinophiles, and without any changes in basophils [17].

No significant increase in leukocytes, however, was reported by Stanek et al. [6], although they also observed a significantly increased percentage of monocytes in healthy individuals after a series of 10 two-minute long WBC sessions at −120°C.

No significant change in the number of leukocytes was also found in professional rugby players by Banfi et al. [12], but perhaps the small number (only five) of single daily WBC treatments was insufficient to induce changes in this aspect. In other studies, in which field hockey players were subjected to WBC two times a day for nine consecutive days, no changes were observed in the amount and percentage of white blood cells, either directly or one week after the last WBC treatment [18]. Significant changes in the white blood cell system (with the exception of the percentage of basophils) were observed in professional tennis players, subjected to 10 WBC treatments (2 daily treatments for 5 days) [19] at the end of the competitive season.

Lombardi et al. (15) observed a small but statistically significant reduction in the number of leukocytes in rugby players after 14 WBC treatments (2 times daily for 7 days). At the same time they observed no changes in the amount and proportion of different types of white blood cells, suggesting that “the WBC had no effect on the immune system, neither activating nor inhibiting it.”

In one very recent study with 10 long distance runners and 10 non-athletes subjected to 12 WBC sessions 3 times a week, significant decreases were noted in white blood cells and neutrophils in the athletes but not in the non-athletes, whereas lymphocytes, monocytes, eosinophils, and basophils remained unchanged both in the athletes and non-athletes. According to the authors it could be supposed that the immune system in athletes is more sensitive to the extreme environmental conditions compared to non-athletes [29].

It seems that on the basis of our studies and the observations of other authors, the impact of extremely low temperatures on the white blood system cannot be unambiguously determined. In healthy non-sporting people one can talk about a mobilization of the white blood cells which may have a positive effect on the immune mechanism of the body. Athletes require further well-designed studies on the effects of WBC on the white blood system, taking into account the individual fractions of leukocytes.

### Platelets

Platelets play an important role in various stages of blood coagulation, and are also involved in the body's immune processes, wound healing and defense of the organism against some bacteria [33], [34]. The production of platelets may depend on various factors, including IL-3 and to a lesser extent, erythropoietin [24].

In this current study we found no changes in the number of blood platelets induced by the WBC treatments, regardless of the number of treatments. The platelet count was not affected by changes in cytokines (EPO and IL-3).

On the basis of our present research and other works [12], [14], [15], [16], [29] it can be concluded that WBC does not affect the number of platelets, regardless of the training status of respondents or the number of WBC treatments.

### EPO and IL-3

To our knowledge, there are no reports on the influence of cryogenic temperatures on the level of erythropoietin and interleukin-3, i.e. cytokines which have a significant effect on hematopoiesis.

Erythropoietin is the first identified hematopoietic factor that regulates survival, proliferation and differentiation in erythrocytic progenitors and the maturation of erythrocytic precursors. It improves the survival of circulating red blood cells and inhibits their degradation – eryptosis [23]. EPO acts as a hormone, and at the same time it is considered to be a hematopoietic cytokine [35], [36]. However, the role of EPO goes far beyond the hematopoietic activity. Erythropoietin acts also as a cardio-, neuro-, hepato- and nephroprotective agent, protecting the cells of these tissues against hypoxia. It also protects endothelial cells, smooth muscle and cardiomyocytes against apoptosis, modulates immune responses, has an anti-inflammatory effect and stimulates angiogenesis [36], [37], [38], [39].

After 10 WBC treatments we observed a significant 4.5% increase in the concentration of EPO. The concentrations of the hormone increased by 10% after 20 and 30 treatments compared to baseline, while no changes in the concentration of this hormone were observed in the control group. The main factor stimulating the synthesis of the hormone is hypoxia. In the period of 60–120 minutes from the stimulus, EPO level in peripheral blood increased, reaching a maximum concentration after about 20 hours [40]. After altitude training, Chapman et al., 1998 [42] observed an increase in EPO concentration by 34–52%, and in states of severe anemia or hypoxia levels of this hormone can be increased up to 1000 times [41]. Thus, the stimulation of the synthesis of EPO we observed was small and did not result in a significant stimulation of the bone marrow. Accordingly, WBC cannot be regarded as an unethical activity that increases erythropoiesis in athletes.

Interleukin-3 is a cytokine which interacts with other cytokines, acting on various hematopoietic cell lines [24], [43]. The effects of IL-3 depend on the presence of other cytokines such as IL-5, GM-CSF (granulocyte-macrophage colony stimulation factor), G-CSF (granulocyte-colony stimulation factor), thrombopoietin and erythropoietin. IL-3 interacts with EPO, stimulating the development of erythroid colonies [24], [25]. In cooperation with erythropoietin, IL-3 stimulates the proliferation and differentiation of early erythroid progenitor cells. In patients after chemotherapy, IL-3 was observed to have a stimulatory effect on neutrophils, reticulocytes and platelets [24]. Il-3 also modulates the development of regulatory T cells, enhances the level of inflammatory cytokine IFN-γ and stimulates migration and proliferation of vascular smooth muscle cells [44], [45]. Despite such diverse activities, cytokine has not been previously studied in athletes or individuals subjected to WBC.

In the present study IL-3 concentrations in blood decreased following the series of WBC treatments. The level of this cytokine reduced after 10 treatments, compared to the control group. However, the greatest decrease compared to baseline was observed after 30 WBC treatments. On the basis of our results it cannot be clearly evaluated what the consequences might be of the reduced level of this interleukin for the examined blood counts.

Study limitation. For a full explanation of the observed changes in hematological parameters as well as in EPO levels, a measurement or at least a calculation of plasma volume are necessary. Unfortunately, using changes in hemoglobin concentration and hematocrit for calculating changes in plasma volume in this study would be confusing because of hemolysis of erythrocytes observed after a series of WBC. The topic, however, is extremely interesting since there is only one report concerning changes of plasma volume after whole-body cryostimulation calculated on the base of Hb and Ht [15]. The changes observed by Lombardi et al. were minimal (+1.51%), however, the range of the changes spanned from −6.24% to 8.75%. It seems that further, carefully designed studies are needed for accurate assessments of changes in plasma volume after WBC.

In summary, the reduced values of the erythrocytic system, an increase in plasma hemoglobin and bilirubin with a simultaneous decrease in haptoglobin concentrations observed after 10 and 20 WBC treatments, may be the result of increased hemolysis of erythrocytes in young healthy men rather than changes in plasma volume. At the same time a small increase in erythropoietin concentration stimulates erythropoiesis, resulting in the red blood cell count and hematocrit and hemoglobin levels returning to normal after 30 WBC treatments. Therefore, in spite of this small rise in EPO concentration, WBC could not be regarded as an unethical method used for stimulation of the erythrocytic system.

The increase in the number of leukocytes, the percentage of lymphocytes and granulocytes observed after 10 and 20 WBC treatments may indicate a moderate leukocyte mobilization, although transient after the next ten treatments.
